# Green Chemistry Based Gold Nanoparticles Synthesis Using the Marine Bacterium *Lysinibacillus odysseyi* PBCW2 and Their Multitudinous Activities

**DOI:** 10.3390/nano12172940

**Published:** 2022-08-26

**Authors:** Tijo Cherian, Debasis Maity, Ramasamy T. Rajendra Kumar, Govindasamy Balasubramani, Chinnasamy Ragavendran, Suneelkumar Yalla, Raju Mohanraju, Willie J. G. M. Peijnenburg

**Affiliations:** 1 Department of Ocean Studies and Marine Biology, Pondicherry University—Port Blair Campus, Port Blair 744 112, Andaman and Nicobar Islands, India; 2 Aquatic Animal Health and Environment Division, ICAR-Central Institute of Brackishwater Aquaculture, Chennai 600 028, Tamil Nadu, India; 3 ETH Zürich—Department of Biosystems Science and Engineering ETH (D-BSSE ETH Zürich), Mattenstrasse 26, 4058 Basel, Switzerland; 4 Advanced Materials and Research Laboratory (AMDL), Department of Nanoscience and Technology, Bharathiar University, Coimbatore 641 046, Tamil Nadu, India; 5 Department of Biotechnology, Division of Research & Innovation, Saveetha School of Engineering, Saveetha Institute of Medical and Technical Sciences (SIMATS), Saveethanagar, Chennai 602 105, Tamil Nadu, India; 6 Department of Biotechnology, School of Biosciences, Periyar University, Salem 636 011, Tamil Nadu, India; 7 Department of Cariology, Saveetha Dental College and Hospitals, Saveetha Institute of Medical and Technical Sciences (SIMATS), Chennai 600 077, Tamil Nadu, India; 8 Institute of Environmental Sciences (CML), Leiden University, P.O. Box 9518, 2300 RA Leiden, The Netherlands; 9 National Institute of Public Health and the Environment (RIVM), Center for Safety of Substances and Products, P.O. Box 1, 3720 BA Bilthoven, The Netherlands

**Keywords:** nanotechnology, gold nanoparticles, *Lysinibacillus odysseyi*, antioxidant, antibacterial, dye degradation

## Abstract

Green chemistry has paved an ‘avant-garde avenue’ in the production and fabrication of eco-friendly stable nanoparticles employing the utilization of biological agents. In the present study we present the first report on the potential of the marine bacterium *Lysinibacillus odysseyi* PBCW2 for the extracellular production of gold nanoparticles (AuNPs). Utilizing a variety of methods, AuNPs in the cell-free supernatant of *L. odysseyi* (CFS-LBOE) were identified and their antioxidant, antibacterial, and dye-degrading properties were examined. The visual coloring of the reaction mixture to a ruby red hue showed the production of LBOE-AuNPs; validated by means of XRD, TEM, SEM, XRD, DLS, TGA, and FT-IR analysis. Additionally, the 2,2-diphenyl-1-picrylhydrazyl technique and the well diffusion assay were used to examine their dose-dependent antioxidant and antibacterial activity. These biogenic LBOE-AuNPs showed 91% dye degradation efficiency during catalytic reduction activity on BTB dye, demonstrating their versatility as options for heterogeneous catalysis.

## 1. Introduction

The development of nanotechnologies has mainly focused on establishing the synthesis and fabrication of nanomaterials through physical and chemical methods. These methods include hydrothermal procedures, gamma irradiation, photochemical or chemical reduction, microwave irradiation, and thermal breakdown processes [1,2,3]. These processes have some limitations. For instance, (a) the hydrothermal approach involves the use of expensive autoclaves, specific safety precautions during the reaction, and the inability to observe the reaction process in the dark [4]; (b) chemical reduction procedures have been found to be effective, but they call for the use of toxic reagents such as hydroquinone, hydrazine, ascorbic acid, dimethylamine borane, alkaline solution, Tollen’s reagent, etc. [5]; (c) high-frequency gamma radiation or variable-wavelength UV light are required for photochemical synthesis; (d) ultrasound-assisted nanoparticle production requires ultrasonic irradiation, an energy-intensive technique with limited scalability [6]. These synthetic protocols have resulted in the scaling up of nanomaterials with the desired structural properties, but their synthetic methods have been quite expensive, time-consuming, and tedious [4,7]. Additionally, some toxic chemicals have also been absorbed on the surface of synthesized nanomaterials, limiting their applications in biological systems and in different environments. Therefore, there is an urgent need for the development of environmentally benign nanostructures through thea doption of simple protocols using biological species in either an intracellular or extracellular mode of reaction [8,9,10]. Regarding the aforementioned restrictions, metal nanoparticles produced through green biosynthesis are more cost-efficient, non-toxic, and environmentally benign. This is a simple, quick, one-step technique that can be permeable and is appropriate for large-scale manufacturing. In this method, metal salts are converted to particles and then stabilized using a biological template (e.g., microbes, phyto-extracts, biomaterials, biological polysaccharides, etc.) [11,12,13]. The synthesis of nanoparticles is usually triggered by biological compounds present in the functional extracts of microbes or plants [14,15,16]. In nature, microbial species of bacteria, fungi, cyanobacteria, yeast, algae, and actinomycetes are reported to produce inorganic nanoparticles [15]. Microorganisms are thought of as nano factories that hold great promise as economical tools, which are environmentally benign and free of harsh and poisonous chemicals as well as energy requirements for the synthesis of nanoparticles. Due to the presence of several reductase enzymes, microorganisms have the capacity to collect and detoxify heavy metals. The reduction of metal salts into nanoparticles requires the help of these reductase enzymes. In the creation of nanoparticles, proteins, reducing cofactors, metal-resistant genes, enzymes, and organic materials play essential roles as reducing and capping agents [17]. Presently, microbially mediated nanomaterials with different degrees of patterns and compositions are exceptionally limited and restricted to some metals and metal-oxides, and all synthesized by terrestrial microbes [16]. With the intense scrutiny and investigations on the fabrication of nanomaterials in terrestrial microbial forms, marine counterparts are now in the limelight of scientific studies and experimentations. Marine microbes have been studied in relation to their dynamics from surface waters to the seafloor for over a decade, and in this context, they are supposed to be keystone species in the reduction of inorganic elements in the sea, due to their underexplored unique and distinctive metabolic pathways [18].

Many marine microorganisms are important in the fabrication of nano-structured mineral crystals and metallic nanomaterials with striking properties such as shapes, sizes, arrangements, and compositions similar to chemical-based ones. Investigations into the production of diverse metal NPs of silver, gold, zinc, titanium, copper, alginate, and magnesium have been conducted using a variety of microorganisms, particularly bacteria and fungi [19]. According to the literature, actinomycetes, bacteria, fungi, and viruses may biosynthesize metal nanoparticles (NPs), including silver, gold, silver-gold alloy, tellurium, platinum, copper, zinc, selenium, palladium, silica, zirconium, quantum dots, titanium, and magnetite [20,21,22]. The marine fungus *Penicillium fellutanum* (isolated from coastal mangrove sediments) was reported as an active fungus in bioreducing the metal solution of AgNO_3_ into silver nanoparticles in an extracellular context [23]. In this line, Gomathi et al. [24] demonstrated the extracellular bio-fabrication of silver nanoparticles and lipid/silver nanocomposites by *Thraustochytrium* species. Rammohan and Balakrishnan [25] reported the formation of polydispersed intracellular silver nanoparticles with sizes ranging from 20 to 100 nm, using a novel strain of *Pseudomonas* sp. 591786. Similarly, Seshadri et al. [26] reported the production of sulfur-rich peptide-capped lead sulfide nanoparticles by the marine yeast *Rhodosporidium diobovatum*. Interestingly, a single-cell protein producer, *Spirulina platensis*, was also capable of bio-reduction and synthesizing protein-capped gold, silver, and bimetallic nanoparticles [27]. The extract of the marine cyanobacterium *Oscillatoria willei* NTDM01 was used to stabilize silver nanoparticles with protein [28]. The benefits of stabilized cadmium sulphide (CdS) nanoparticles (5 nm in size) by C-phycoerythrin (C-PE) extracted from *Phormidium tenue* NTDM05, a marine cyanobacterium, were reported for the first time using bio-labeling experiments [29]. The microbial species of *E. coli* and *Bacillus subtilis* (AuNPs) [30,31], *Lactobacillus casei* and *B. cereus* (AgNPs) [32,33], *Magnetospirillum magnetotacticum* and *Aquaspirillum magnetotacticum* (Fe_2_O_3_-NPs) [34,35], *Shewanella oneidensis* (UO_2_-NPs) [36], *Klebsiella aerogenes* and *E. coli* (CdS-NPs) [37,38], and *Aspergillus flavus* (SeNPs) [39] have been reported as proficient producers of nanostructures.

Due to the ability to change the particle size and hence regulate its properties, colloidal nanoparticles have become increasingly important in recent years. With sizes ranging from 1 nm to more than 100 nm, they can be easily synthesized in a variety of shapes, including spheres, rod-like particles, core-shell particles, and many others [40,41]. They also have a wealth of shape- and size-dependent optical and electronic properties, as well as the capacity to absorb light in the visible spectrum. Gold nanoparticles (GNPs) have emerged as the most stable metal nanoparticles due to their distinct optical characteristics, resulting from their surface plasmon resonance [42]. Due to AuNPs’ extraordinarily high absorption coefficients, optical detection techniques can be more sensitive than techniques using traditional dyes. It has been advantageous to employ AuNPs’ strong absorption for colorimetric analyte detection, either by causing AuNPs to aggregate in the presence of particular analytes or by monitoring changes in the environment’s refractive index brought on by the adsorption of bio-analytes [43]. Other intriguing optical features with significant potential for bio-diagnostic assays are provided by the significant amplification of the electromagnetic field at the surface of AuNPs caused by their interaction with electromagnetic radiation [44]. Despite the instigation of such breakthroughs, exhaustive, comprehensive work is urgently required in the domain of marine-derived gold nanobiotechnology. Some of the potential challenges in nano-applicable proposals are related tot he mechanisms required for making nanoparticles from marine sources and their biological activities.

The emergence of novel drug-resistant microorganisms is the main obstacle faced by medical practitioners. Therefore, the creation of novel medications is required to treat a variety of disorders. Applications of NPs in medicine provide a variety of benefits, including early detection systems, NP-based imaging diagnosis, and therapy for many diseases brought on by drug-resistant microorganisms [20,45]. Because of their antibacterial and immunological properties, the development of nanotechnology and the techniques utilized to create nanocomposites/NPs have also transformed the world of biomedicine [46]. The biosynthesis of NPs using various microbes and plants makes them attractive candidates for the manufacturing of new antibacterial nanomaterials [47,48]. Pathogenic Gram-negative bacteria such *E*. *coli*, *Klebsiella pneumonia*, *Salmonella typhimurium*, *Pseudomonas aeruginosa*, *Proteus mirabilis*, *Shigella dysenteriae*, *Enterobacter aerogenes*, and *Citrobacter* sp. exhibit highly antibacterial activity towards Au-NPs. Additionally, biosynthesized Au-NPs have exhibited antimicrobial activity against Gram-positive pathogenic bacteria such *Staphylococcus epidermidis*; *Staphylococcus aureus*, including MRSA; *Streptococcus pyogenes*; *Enterococcus faecalis*; and *Bacillus subtilis* [45,49,50].

Furthermore, due to their capacity to reduce substances quickly and effectively, metal nano catalysts have received greater attention in recent years for their use in dye degradation. The organic contaminants and dyes/pigments found in wastewater discharged by a variety of industries, such as textiles, paper, plastic, wood, leather, pharmaceutical, agricultural, and cosmetics, have grown to be a serious problem for environmental protection [51]. The bulk of dyes and pigments are aromatic chemical complexes with chromophoric functional groups, which can absorb visible light between 350 and 700 nm [52]. Wastewater containing these toxic chromophores could impair human health, in addition to threatening aquatic life [53]. Therefore, such dyes/pigments must be degraded from wastewater before being discharged from industry to the environment. Today, a variety of wastewater treatment methods are frequently used [54,55]. Some of them, though, have proven to be costly and ineffective. As an illustration, the coagulation and flocculation process requires a lot of dangerous chemicals (including limes, alums, ferric salts, etc.) [56]. Adsorption procedures often involve heavy metal removal and produce secondary sludge [57]. Reverse osmosis is a challenging method for controlling membrane fouling in ultra-filtration technology [58]. Other conventional methods, such electrochemical ones, have a slew of disadvantages as well, including high energy needs, a need for chemical reagents, and high operational expenses for tanks and dosage equipment [59]. Photocatalytic reduction and Fenton-like degradation can now be used to remediate these pollutants [55,60]. Their extensive use is constrained, though, by a number of important limitations (such as the need for UV light, a small band gap, and a low pH operating range to prevent catalytic precipitation) [11,61]. Therefore, it has become vital to find appropriate, affordable, and ecologically acceptable wastewater treatment solutions.

*Lysinibacillus odysseyi* is a Gram-positive, catalase-positive, oxidase-negative rod that was named after the Mars Odyssey spacecraft from which it was isolated for the first time. The species *L. odysseyi* is obtained from the tidal flat sediment of the Shinduri sand dunes at Tae-An, Yellow Sea coast, Republic of Korea [62]. This study was the first attempt to use CFS-LBOE as a reducing agent in the marine bacterium-mediated extracellular synthesis of AuNPs. The modalities of characterization were also classified by employing varied technological mechanization approaches. Interestingly, this paper also serves as the first report on the antioxidative, antibacterial, and dye degradation properties of *L*. *odysseyi* biofunctionalized AuNPs.

## 2. Materials and Methods

### 2.1. Collection and Processing of the Sample

A seawater sample was collected from the coast of Chidiyatapu, South Andaman (11°29′24.36″ N, 92°42′25.24″ E) using a sterile polyethylene bottle and transported to the laboratory under sterile conditions. One milliliter of seawater was aseptically transferred to a sterile conical flask containing 99 mL of filtered sterile seawater. The serial dilutions were carried out up to 10^−6^ and 0.1 mL was plated onto the successive Zobell Marine agar plates by means of the spread-plate technique and incubated for 24 h at 35 °C. After incubation, single and discrete isolated colonies were streaked again to obtain the pure isolates from a single colony, and their morphology was observed under a microscope. The isolates were maintained as slants, stabs, and in 10% glycerol cultures for further analysis.

### 2.2. Phenotypic and Genotypic Characterization

A series of biochemical tests were undertaken in order to identify isolates based on the phenotypic characteristics listed in Bergey’s *Manual of Systematic Bacteriology* [63]. Furthermore, the Gram staining method was performed for the isolates. Growth optimization studies at varying salt concentrations and temperatures were performed. The parameters with optimal growth were inferred by using the Ident ax Bacterial Identifier (Software Version 1.2) with an identical score of more than 95% [64]. The genotypic analysis was carried out via16s rRNA gene amplification using universal consensus primers 27F and 1492R [65]. A total of 50μLof reactions were carried out, and the amplified products were chosen for sequencing. The sequences that were found were compared and sent to NCBI to obtain an accession number.

### 2.3. Chemicals

Chloroauric acid (HAuCl_4_·3H_2_O), hydrochloric acid (HCl), sodium hydroxide (NaOH), antibiotic susceptibility discs, nutrient broth (NB), Muller–Hinton broth (MHB), and agar (MHA) were procured from HiMedia Laboratories (India) Ltd., Mumbai. Ethanol was purchased from Changsha Hong Sheng Fine Chemicals Co., Ltd., Suzhou, China.

### 2.4. Preparation of Bacterial Extract

The bacterium *Lysinibacillus odysseyi* was grown on Zobell marine broth under culture conditions (30 °C; constant shaking at 150 rpm for 24 h). Following incubation, the biomass was subjected to centrifugation (conditions: 10,000 rpm, 4 °C, 15 min). Both the supernatant (ST) and cell biomass were rescued for the determination of enzyme localization, i.e., to qualify whether the enzyme was intracellular or extracellular. The resulting cell biomass was washed twice in sterile phosphate buffer (pH 7, 0.05M) via centrifugation (6000 rpm). The pellet formed was dissolved in 50 mL sterile distilled water, followed by sonication at 5 min with 35 s pulse. The resulting solution was centrifuged (10,000 rpm, 4 °C, 15 min) and the resultant cell free supernatant (CFS) was preserved for further analysis [66].

### 2.5. Synthesis, Optimization, and Yield of LBOE-AuNPs

For thorough and methodical investigations of biofunctionalized gold nanoparticles (AuNPs) using *Lysinibacillus odysseyi* extract (LBOE) with minor modifications, process optimization and standardization were pursued [67,68]. The formation of LBOE-AuNPs was appraised under the following conditions: concentrations of cell-free supernatant (CFS) of LBOE (10%, 20%, 30%, 40%, and 50%), different pH values (3, 5, 7, 9 and 11), the concentration of HAuCl_4_ (0.5, 1.0, 1.5, 2.0, 2.5, and 3.0 mM), the ratio of metal solution to CFS-LBOE, and the temperature range (20, 40, 60, 80, 100 °C). The pH variation was regulated by altering the reaction mixture with NaOH accordingly. The stability of the synthesized LBOE-AuNPs was monitored for about 6 months through the measurement of the spectral readings [69,70].

### 2.6. Characterization of Gold Nanoparticles

The physicochemical characteristics of LBOE-AuNPs were determined using the following analytical techniques: UV-visible spectrophotometry (Cintra 101, GBC Scientific Equipment Ltd., Braeside, Australia); X-ray diffractometry (XRD, Rigaku Corporation, Tokyo, Japan); TEM (transmission electron microscopy); SEM (scanning electron microscopy) (JEOL, Tokyo, Japan); EDX (energy-dispersive X-ray) analyses (Oxford Instruments, INCAx-sight, Killeen, TX, USA); dynamic light scattering (DLS) (nanoparticle size analyzer, VASCO-3, Cordouan Technologies, Pessac, France); zeta potential analysis (Zeta Sizer Nano ZS-90 Malvern Instruments, Malvern, UK); thermogravimetric analysis (TGA) (thermogravimetric system, Perkin-Elmer), and Fourier transform infrared spectroscopy (FT-IR) (FT-IR spectrometer Spectrum Two, Perkin Elmer Life and Analytical Sciences, Waltham, MA, USA). All characterization techniques, along with operational conditions, were implemented according to previous reports [7,10,13].

### 2.7. Purification and Sterilization of AuNPs

The biosynthesized LBOE-AuNPs were fabricated using microbial secreted bio-molecules of varied functionalities, excess metallic salt HAuCl_4_, and other impurities that may influence biological activity and efficacy. The LBOE-AuNPs were washed thrice with sterile double distilled water via centrifugation (15,000 rpm; 30 min) and the obtained pellet was re-suspended in sterile double-distilled water for future biological applications [71]. In order to avert any contamination in the biosynthesized AuNPs before invitro studies, they were suspended in 1X phosphate buffer saline (PBS) and subjected to tyndallization, in which the AuNP solution was superficially heated for 30 min over four consecutive days. The resultant samples were incubated at room temperature for 24 h and finally freeze-dried [72].

### 2.8. In Vitro Antioxidant Assays

#### 2.8.1. DPPH Radical Scavenging Activity

The electron-donating capacity (or hydrogen atoms) of LBOE-AuNPs was examined based on the color bleaching sensitivities of 2,2-diphenyl-1-picrylhydrazyl (DPPH) in methanol [73]. About 50 μL LBOE-AuNPs of different concentrations (20, 40, 60, 80, and 100 µg/mL) were mixed separately with 450 μL Tris–HCl buffer (pH = 7.4) and 1 mL of methanolic DPPH solution (0.1 mM) and were incubated at room temperature in the dark for 30 min, measuring their absorbance values at 517 nm, respectively. The standard (ascorbic acid) and blank (methanol) were used. The inhibitory concentration at which 50% (IC_50_ value) of DPPH free radicals were scavenged by LBOE-AuNPs was evaluated. The percentage of inhibition of DPPH radicals was calculated by means of the following equation (Equation (1)):

(1) $$\mathrm{Inhibition}\;\left(\%\right)=(\left(A\;\mathrm{blank}-B\;\mathrm{sample}\right)/A\;\mathrm{blank})\times 100$$

where A blank refers to the absorbance of the control reaction and B sample refers to the absorbance of the test compound.

#### 2.8.2. Reducing Power Assay

The reductive ability of LBOE-AuNPs, i.e., the transformation of Fe^3+^ ions into Fe^2+^ ions, was investigated using the techniques of Oyaizu [74] and Liu and Yao [75]. The LBOE-AuNPs (20 to 100 µg/mL; diluted with 1.0 mL of double distilled water) were mixed with 2.5 mL of sodium phosphate buffer (0.2 M; pH 6.6) and 2.5 mL of potassium ferricyanide (K_3_Fe(CN)_6_; 1%). The sample combination was incubated at 50 °C for 20 min, before the addition of 2.5 mL of trichloroacetic acid (TCA; 10%). The resultant mixture was centrifuged at 3000 rpm for 10 min. The absorbance at 700 nm was measured after mixing the top organic layer (2.5 mL) with sterile double-distilled water (2.5 mL) and 0.5 mLof ferric chloride (FeCl_3_; 0.1%). A rise in absorbance implies that the reducing power is strong. The standard and blank solutions were ascorbic acid and sodium phosphate buffer, respectively. Equation (1) was used to calculate the percentage of reduction (Fe^3+^ to Fe^2+^ ionic state).

### 2.9. Evaluation of Antibacterial Activity of LBOE-AuNPs

#### 2.9.1. Maintenance of Pathogenic Strains

Four pathogenic strains, namely, *Aeromonas hydrophila* IDH1585, enterotoxic *Escherichia coli* serotype 0115, *Vibrio cholera* MTCC 3905, and *Staphylococcus aureus*, were used for testing antibacterial activity andthese were periodically sub-cultured on Mueller Hinton agar (MHA) and maintained as slants/stabs (4 °C) and 10% glycerol stocks (−20 °C).

#### 2.9.2. Minimal Inhibitory Concentration (MIC) of LBOE-AuNPs

Todetermine the MIC of LBOE-AuNPs, the broth micro-dilution method was performed [76,77,78]. Different concentrations of LBOE-AuNPs (10, 20, 50 and 100 µg/mL) were tested on a bacterial suspension (10^8^ CFU/mL) and control tubes were maintained at 37 °C for 24 h. The optical density of the culture broths was measured to be 620 nm, and the MIC was found.

#### 2.9.3. Minimal Bactericidal Concentration (MBC) of LBOE-AuNPs

For MBC determination, aliquots (20 µL) from all culture tubes used in the MIC assay were seeded, cultured on nutrient agar plates, and incubated for 24 h at 37 °C [77,79].

#### 2.9.4. Time-Kill Curve Assay

The time-kill assay was employed to determine the antimicrobial effect, which is contingent on the aspects of time or concentration dependence [80]. The effects of LBOE-AuNPs on the bacterial growth curve pattern in liquid nutrient broth were examined following the National Committee for Clinical Laboratory Standards (NCCLS) [81,82]. A freshly grown culture with a cell density of 2 × 10^8^ CFU/mL was used. One milliliter of cell suspension was added to 100 mL of nutrient broth supplemented with 0, 10, 20, 40, 60, 80, and 100 µg/mL of LBOE-AuNPs and incubated at 37 °C under constant shaking conditions (150 rpm). The O.D. of bacterial growth was measured at 620 nm every two hours for up to 12 h.

#### 2.9.5. Agar Well Diffusion Assay

The antibacterial activity of microbial extracts was evaluated using the agar well diffusion method [83,84]. For example, 0.1 mL of bacterial inoculum (cell density of 2 × 10^8^ CFU/mL) was homogeneously spread on the Mueller–Hinton agar plates, and the wells were made using a cork-borer. Subsequently, different amounts of LBOE-AuNPs (20, 40, 60, 80, and 100 µg/mL) were added to the pre-cut wells, followed by the incubation of plates at 37 °C for 24 h. The zone of inhibition was measured using the antimicrobial zone scale (HiMedia, Mumbai, India).

#### 2.9.6. Effect of LBOE-AuNPs on the Bacterial Biofilm

The anti-biofilm efficacy of LBOE-AuNPs was assessed against two strains by means of the crystal violet (CV) assay with slight modifications [85]. Starter cultures (100 μL) of *E. coli* and *Staphylococcus aureus*, grown overnight in nutrient broth (cell density ~10^8^ cells/mL), were seeded into the 96-well microtiter plate (Corning, USA). A predetermined volume (100 μL of culture medium) was amended with variable concentrations (20–100 μg/mL) of LBOE-AuNPs into the respective wells. A control setup of untreated bacterial cells in nutrient broth was run as a parallel positive control, and the plates were incubated at 37 °C for 24 h. The loosely remaining cells were washed out thrice with autoclaved seawater + phosphate buffer saline (PBS) in 1:1 and a CV solution (200 μL, 0.25%) dispensed in all the wells and incubated at 37 °C for 30 min. The unbound CV was then washed with seawater + phosphate buffer saline (PBS) and the wells were air-dried. The CV bound to bacterial cells was then dissolved in 250 μL ethyl alcohol (95%) and the absorbance values (OD) were measured at 620 nm.

### 2.10. DNA Cleavage Assay

The cleavage pBR322 vector DNA treated with LBOE-AuNPs was examined by means of a slightly modified protocol [86]. Aliquots (50 μL) of HAuCl_4_ and LBOE-AuNPs (20–100 µg/mL) were added separately to the vector DNA (1μL; concentration 0.5 μg/mL) in TE buffer (10 mM Tris–HCl, 0.1 mM EDTA, pH 7.4) and incubated for 24 h at 37 °C under dark conditions. Subsequently, gel electrophoresis was carried out using 20 μL HAuCl_4_ and LBOE–AuNPs–bacterial DNA mixtures were loaded and subjected to 1% agarose gel electrophoresis. After electrophoresis, the gel was viewed and photographed using a gel documentation system with a UV light trans-illuminator [87,88].

### 2.11. Mechanism of Action of LBOE-AuNPs on Microbial Cells

#### 2.11.1. Measurement of Cellular Leakages

##### Protein Leakage Assay

The estimation of the leakage of protein constituents was ascertained by means of the previously developed protocol [89]. The bacterial cells were treated and incubated with LBOE-AuNPs (MIC concentrations) for 3–6 h, followed by centrifugation (6000 rpm; 15 min). For each subsequent sample, the supernatant (200 µL) was mixed with Bradford reagent (800 µL) and incubated for 10 min. BSA, which is made from the blood of cows, was used as a standard to measure the optical density at 595 nm.

##### Nucleic Acid Leakage Assay

The estimation of nucleic acid leakage was determined by means of an earlier reported standard protocol with slight modifications [90]. Aliquots of bacterial cultures were incubated with LBOE-AuNPs (MIC concentrations) at the time intervals of 3–6 h and filtered through Millex-GS syringe filters (Millex-GS, Madrid, Spain) with the following dimensions: a diameter of 25 mm and a pore size of 0.2 µm. The absorbance values were read at 260 nm.

### 2.12. Photocatalytic Degradation of Dye Bromothymol Blue by LBOE-AuNPs

The protocols of photocatalytic degradation by LBOE-AuNPs were performed as described in an earlier report [91]. The dye, BTB (bromothymol blue), was taken as a demonstrative water pollutant for the evaluation of the photocatalytic activity by LBOE-AuNPs. The dye BTB solution (2 × 10^−5^ mol/L) was prepared at pH 12 using NaOH (0.1 N) for deep blue coloration. A differential series of LBOE-AuNPs concentrations (50, 100, 150, and 200 µg/mL) was added to 150 mL of aqueous dye solution in separate experimental flasks and stirred using a magnetic stirrer for 60 min under dark conditions. The resulting dye mixtures were then kept under solar irradiation and the dye degradation was elicited by adding 3.0 mL of H_2_O_2._ The absorbance at 616 nm was measured by using 2 mLof dye solutions that had been collected immediately. Every 15 min, the photo-catalytic activity was monitored until the dye was completely degraded into its colorless state. Photo-catalyzed LBOE-AuNPs were collected again via centrifugation and washed twice with distilled water and ethanol (30%, 50%, 70%, and 90%) so they could be used again in the relevant cycles.

### 2.13. Statistical Analysis

All quantitative experimentation was carried out in triplicate experiments. The standard error of the mean was calculated; the results shown in the figures and text represent the mean ± standard deviation calculated using Sigma plot 10.0 version software.

## 3. Results

### 3.1. Extracellular Synthesis of AuNPs

Among the isolated bacterial strains, one bacterial isolate, *Lysinibacillus odysseyi* PBCW2 (MK611701), exhibited the capability to perform Au^3+^ ion reduction, leading to the formation of AuNPs, and this was indicated by the visualization of a change in color from golden yellowish to ruby-red due to the phenomenon of SPR (surface plasmon resonance) observed in bio-synthesized LBOE-AuNPs [92]. The noticeable color change of the reaction mixture to ruby-red, pinkish, or violet is the first visual clue indicating LBOE-AuNPs synthesis [8,9,67,93]. A similar study by Roy et al. [94] reported a color change to deep pink (at 520 nm) in the reaction mixture of a live cell filtrate of *Aspergillus foetidus* + HAuCl_4_, attributable to the high surface plasmon resonance. In the present study, an extracellular synthetic approach was employed in order to purge out the intracellular synthesis-related downstream processing, making the applied methodology economical and straightforward. The mechanism outlining the formation of AuNP remains to be explicated, although previous studies have proposed the role of microbial enzymes that are released extracellularly or secreted as the responsive triggers of the reduction reaction [7,8,10,95].

**Figure 1:**
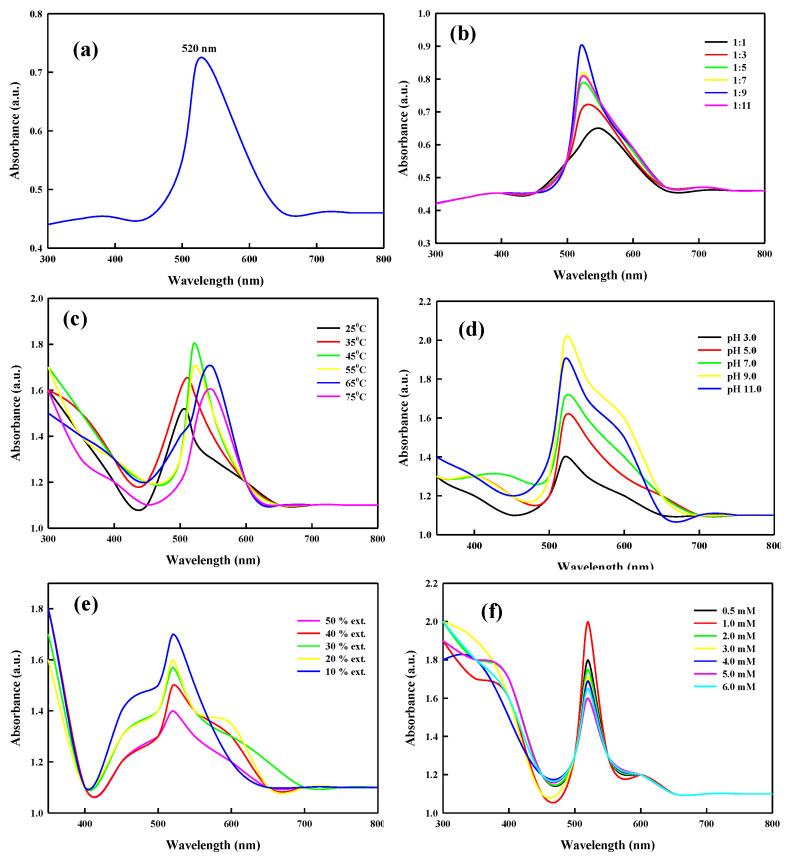
Single plasmon resonance peak of LBOE-AuNPs observed at 520 nm (**a**) and process optimization of LBOE-AuNPs. (**b**) LBOE:HAuCl_4_; (**c**) temperature variation; (**d**) pH variation; (**e**) LBOE concentration and (**f**) variable metal salt concentrations.

The bacterial extract LBOE was used for the reduction of the metal salt solution of HAuCl_4_ into bio-capped AuNPs under the optimum parameters of pH, temperature, concentration of the bacterial extract, strength of the metal salt solution, and ratio of extract to metal solution. The highest rate of AuNP biotransformation was observed under optimized conditions, showing a distinct single peak at 520 nm (Figure 1a). Studies on the extracellular synthesis of AuNPs have reported a spectral peak at 534 nm [9]. Similarly, AuNPs synthesized from *Stenotrophomonas maltophilia* and *Stenotrophomonas* sp. exhibited peak shift absorbance at 530 and 580 nm, respectively [67,96], due to dissimilarities in the shapes and sizes of the synthesized AuNPs [8]. The effect of the ratio of HAuCl_4_:bacterial extract (1:1, 1:3, 1:5, 1:7, 1:9, and 1:11) on AuNP synthesis was comprehensively studied. All ratio concentrations were found to satisfactorily reduce the metal ions into capped AuNPs (Figure 1b), recording spectral absorbance peaks in the range of 510–550 nm, with the most promising AuNP biotransformation observed at the ratio of 1:9 (Figure 1b). Furthermore, the effect of temperature on LBOE-AuNPs synthesis was ascertained (Figure 1c) by exposing the reaction mixture of LBOE and HAuCl_4_ at a ratio of 1:9 to the variables of temperature (25, 35, 45, 55, 65, and 75 °C). The color of the reaction mixture changed from colorless to ruby red within 3 min of the reaction when the reaction temperature was elevated from 25 °C to 45 °C, with the shift observed in the maximum absorption peak from 516 nm to 525 nm.

Furthermore, an increase in the incubation temperature of the reaction from 55 °C to 75 °C intensified the color of the reaction mixture from ruby red to violet-red (peak shift from 525 to 540 nm), indicating an increase in the activation energy and reducing the power of the biological moieties due to the increased thermal effect [97]. Patil et al. [9] reported a sharp broad peak (534 nm) at a temperature of 70 °C, exhibiting the highest absorbance in AuNPs synthesized using an extract from *P*. *haeundaensis* BC74171T. Furthermore, an increase in the reaction temperature enhanced the reaction rate, leading to the formation of small-sized mono-dispersed nanoparticles [8,98,99]. Similar studies based on the microbe-assisted synthesis of metallic nanoparticles indicated an increase in the number of nanoparticles with increasing absorbance [96,100,101]. Furthermore, the bio-reduction of HAuCl_4_ into AuNPs complexed with biomolecules of bacterial extracts in the pH range of 3.0 to 11.0 at 45 °C was evaluated. A rapid biosynthesis of LBOE-AuNPs was detected in the pH range of 7.0–11.0 within 5 min of the reaction (Figure 1d) and the pH of the reaction mixture decreased after the synthesis of LBOE-AuNPs. The absence of flocculation of gold nanoparticles at neutral and alkaline pH values indicates their mono dispersity and stability, whereas a low degree of aggregation was observed at acidic pH values. The rationale for such rapidity in the generation of LBOE-AuNPs is explained as follows. If the bio-molecular part of the peptide (present in the bacterial extract) is amphoteric in nature, its carboxylic groups would undergo protonation when the pH of the solution drops below the isoelectric point (PI) of the peptide part. The prevailing electrostatic repulsion is unable to impart overall stability to the particles, leading to their instability or the absence of particle synthesis. In contrast, if the pH of the solution rises above the PI of the peptide part, the carboxylic groups will be ionized to COO−, resulting in the remarkable binding affinity of Au^3+^ to the negative charge of the former in favor of the reduction of auric ions [102].

**Figure 2:**
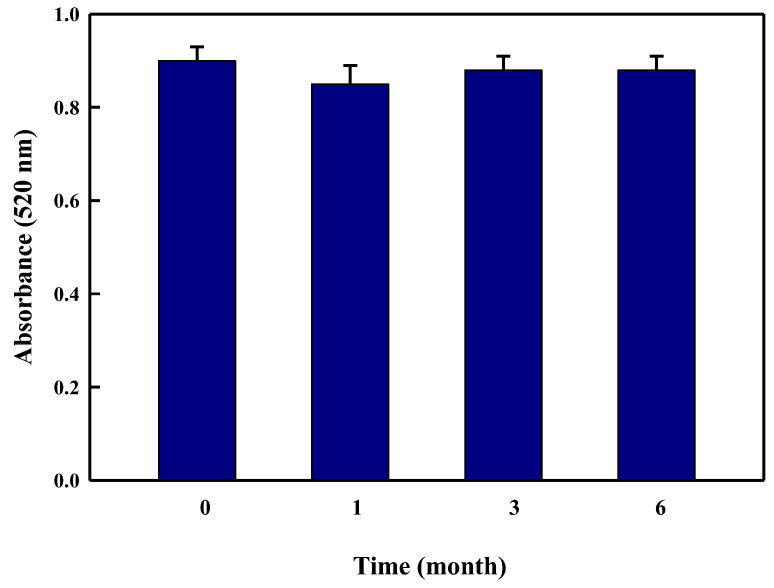
Stability of LBOE-AuNPs.

Furthermore, the effect of the bacterial extract (LBOE) concentration on the reaction rate of LBOE-AuNPs synthesis was examined at a fixed concentration of HAuCl_4_ (1 mM), pH 9.0; temperature 45 °C; and a concentration range of 10%, 20%, 30%, 40%, and 50% (Figure 1e). Based on the spectral readings, we ascertained that the extract concentration of 10% was the most productive in the synthesis of LBOE-AuNPs at 523 nm with increased absorbance intensity, and thus indicative of the complete reduction of auric ions and rapid synthesis of capped LBOE-AuNPs. The rate of LBOE-AuNPs was reported to be similar in concentrations of 20% and 30%, whereas a lower reaction rate was observed in 40% and 50% LBOE concentrations. The formation of small- or large-sized AuNPs is discerned based on a change or shift in peak positions towards the low or high range, respectively [103]. In the present study, the spectral absorbance increased with no shift in the characteristic peak, implying the increased formation of AuNPs with stable morphologies. Similarly, the effect of the metal salt concentration (HAuCl_4_) on the synthesis of LBOE-AuNPs was also studied with concentration ranges of 0.5, 1.0, 2.0, 3.0, 4.0, 5.0, and 6.0 mM at a fixed pH of 9.0, a temperature of 45 °C, and a bacterial extract concentration of 10%. The value of surface plasmon absorbance increased with an increase in the metal salt concentration (Figure 1f). The maximum synthetic activity was observed at 1.0 mM, and similar productive activity was also observed at 2.0 mM. Furthermore, only a slight increase in the generation of AuNPs was observed with concentrations of 3.0–6.0 mM. A stable and characteristic peak at 520 nm was discerned in all the concentrations of HAuCl_4_, with differences only in the color intensity of the reaction, attributable to the SPR and the increased number of AuNPs [92,104]. The bio-fabricated LBOE-AuNPs were found to be relatively stable for about 6 months, with their absorbance values found to be consistent at 520 nm (Figure 2).

### 3.2. Characterization of Synthesized AuNPs

The synthesized LBOE-AuNPs under optimized reaction parameters (pH 9.0, temperature 45 °C, 10% bacterial extract concentration, 1 mM HAuCl_4_ concentration, and bacterial extract: HAuCl_4_ ratio (1:9)) were centrifuged and washed with autoclaved milli-Q water in order to remove non-reactive metal ions and other impurities [71,72,101].

**Figure 3:**
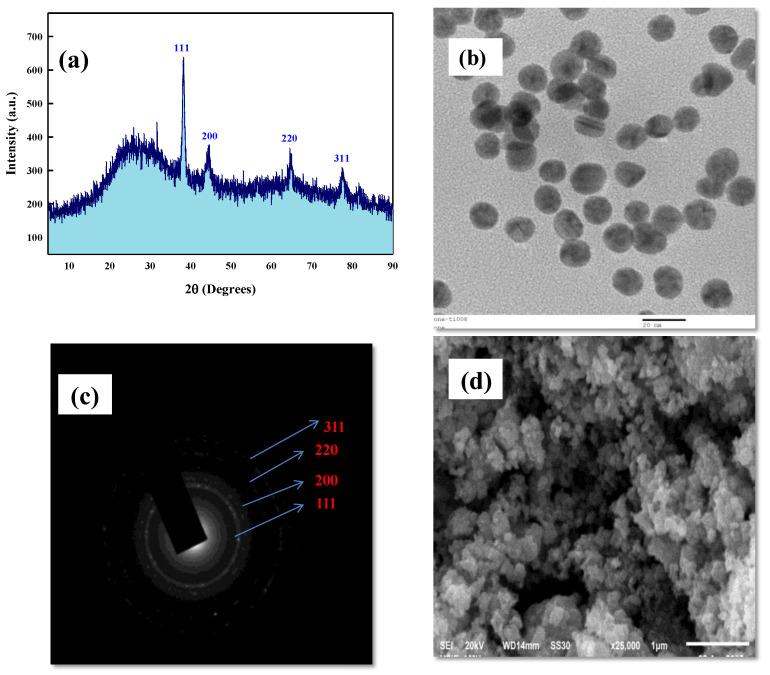
Characterization of LBOE-AuNPs. (**a**) XRD, (**b**) TEM, (**c**) SAED, (**d**) SEM.

XRD (X-ray diffraction) is generally referred to as the prime technique for the complete resolution of tertiary structures of crystalline materials at the atomic to sub-atomic scale [105]. The AuNPs fabricated from bacterial extract sexhibited the major diffractive reflections at 38.2°, 44.3°, 64.2°, and 77.3°, corresponding to the 111, 200, 220, and 311 lattice planes of the crystalline structure (Figure 3a). The broad exhaustive peak at the 2θ value of 38.2° (111) lattice plane of the fcc (face-centered cubic) lattice explicitly indicated that LBOE-AuNPs were made of pure gold. The fraction between the intensities of (200), (220), and (311) reflective diffraction peaks was much lower, suggesting a predominant orientation of the (111) plane [106]. The appearance of these four powerful peaks, which corresponded to LBOE-AuNPs, was consistent with the Bragg’s reflections of gold seen in the diffraction pattern [107]. Interestingly, a halo region was found at the diffraction peak range of 15°–35°, which may have been due to the complexation of ordered and disordered structures of the cellular polymeric molecules containing organic groups with Au^3+^ ions, resulting in the spacing of individual polymeric chains [108,109,110]. A similar result was also reported in an earlier study [111], where yeast *Candida parapsilosis* ATCC 7330-supported gold nanoparticles exhibited a similar XRD pattern of an amorphous halo region. Furthermore, many minor unidentified peaks were reported, which may have been due to the surface association of biomolecules present in the bacterial extract with the biosynthesized LBOE-AuNPs [103]. The intensities and positions of Bragg’s peaks were compared and matched with the standard JCPDS file (File No. JCPDS 4-0783), assuming the average size of LBOE-AuNPs to be 29.28 nm with a cubic structure. These results follow the previously reported studies about the synthesis of gold nanoparticles using macroalgae *Turbinaria conoides* [103], species of *Streptomyces* MBRC-82 and *Nocardiopsis* MBRC-48 [112,113], *Bacillus niabensis* [102], *Klebsiella pneumoniae* [114], *B*. *marisflavi* [115], *Micrococcus yunnanensis* J2 [8,9,93], and *Paracoccus haeundaensis* [9].

Electron microscopy is the most common analytical technique used for the analysis of the size, shape, surface topology, and granularity of nanostructures [7,10,95]. In the present study, TEM micrographs of LBOE-AuNPs (Figure 3b and Figure S1) revealed spherical nanoparticles, with the SAED pattern (Figure 3c) revealing well-defined diffraction spots in the form of rings found in concurrence with the XRD diffractive peaks, indicating the polycrystalline nature of gold. Similar results were also reported in the case of a cell extract of microalga *Tetraselmis suecica*-reduced AuNPs [116] with an average size of 79 nm. The culture medium of *Bacillus marisflavi* produced crystal spherical AuNPs with an average size of ~14 nm within 96 h at room temperature [115]. Jafari et al. [99] reported spherical well-dispersed gold nanoparticles synthesized using *Micrococcus yunnanensis* J2 with a particle size of 53.8 nm. The SEM micrographs (Figure 3d) depicted smaller-sized LBOE-AuNPs with the majority of the AuNPs being similar in shape, with minor to negligible aggregations. Recent scientific findings have reported variable-shaped bacterially synthesized poly dispersed AuNPs [117,118,119,120] in the size range of 15 to 35 nm [8,9,67,93,99], which is consistent with our findings.

**Figure 4:**
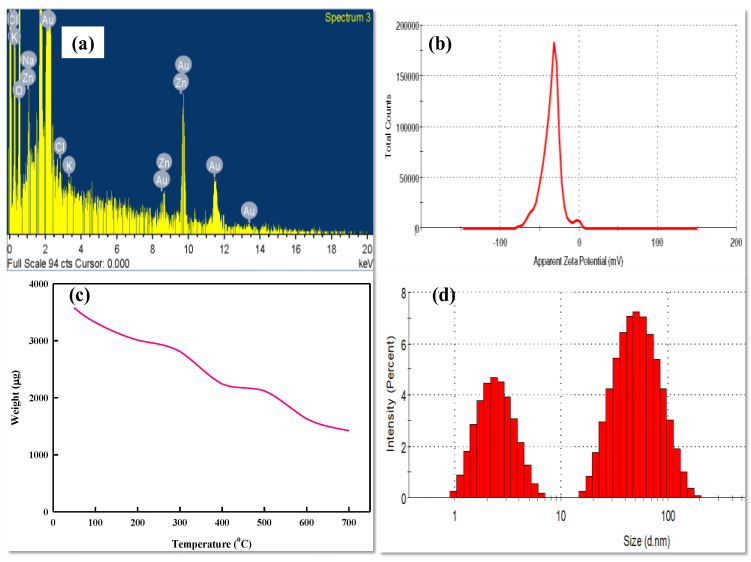
Characterization of LBOE-AuNPs. (**a**) EDX spectrum, (**b**) Zeta potential, (**c**) TGA, (**d**) DLS.

The elemental composition of LBOE-AuNPs ascertained by EDX was found to be gold (74.76%), along with oxygen (20.43%), sodium (1.58%), and chlorine (0.88%) and potassium (0.26%) (Figure 4a). Similar EDX peaks were also reported in the AuNPs synthesized by means of *Streptomyces* sp. MBRC-82 [113] and *Sporosarcina koreensis* DC4 [100]. This result was contested by the non-aggregation of AuNPs after six months of storage stability analysis at room temperature. Nangia et al. [98] evaluated the zeta potential of *Stenotrophomonas maltophilia* biosynthesized AuNPs as −16.7 mV, confirming their stability (Figure 4b). A similar study by Srivastava et al. [121] ascertained the stability of *Escherichia coli* K12-synthesized biogenic AuNPs with their zeta potential value calculated at −24.5 ± 3.1 mV. The zeta potential of *Micrococcus yunnanensis* J2-synthesized AuNPs was found to be −17.6 ± 1.8 mV, suggestive of negatively charged and stable biogenic nanostructures.

The TGA analysis (Figure 4c) revealed that the scale of degradation of organic components tagged on LBOE-AuNPs amounted to 39%, thus affirming the role and mechanism of biological compounds present in the bacterial extract in the surface capping of LBOE-AuNPs. The TGA graph showed that LBOE-AuNPs had three weight losses, detected in the temperature range of 50 °C–700 °C (Figure 4c). Figure 4c shows that the first weight loss, from 60 °C to 180 °C, was caused by water evaporation. The second weight loss, from 340 °C to 400 °C, was caused by the combustion/ignition of organic compounds that were capped on the surface of the AuNPs. The third gradual weight loss, from 580 °C to 700 °C, was caused by the total thermal degradation of organic compounds. These results are in accordance with the earlier reports of Ahmad et al. [122] and Patra et al. [123]. This may be plausibly attributed to a shift in Raman peaks, which are primarily associated with the bond lengths of chemical molecules and the regularity and symmetry of nanoparticles [124,125]. DLS is one of the most extensively used methods for determining the size of nanoparticles (NPs) in colloidal solutions in the nano- and sub-micrometer ranges [126]. The particle size analysis based on DLS revealed the size of LBOE-AuNPs to be between 1–100 nm (Figure 4d). The maximum size range of nanoparticles was found to be 20–100 nm (average particle size = 31.6 ± 9.7 nm), along with a proportion of LBOE-AuNPs also detected between 1–10 nm (average particle size = 5.6 ± 0.7 nm).

**Figure 5:**
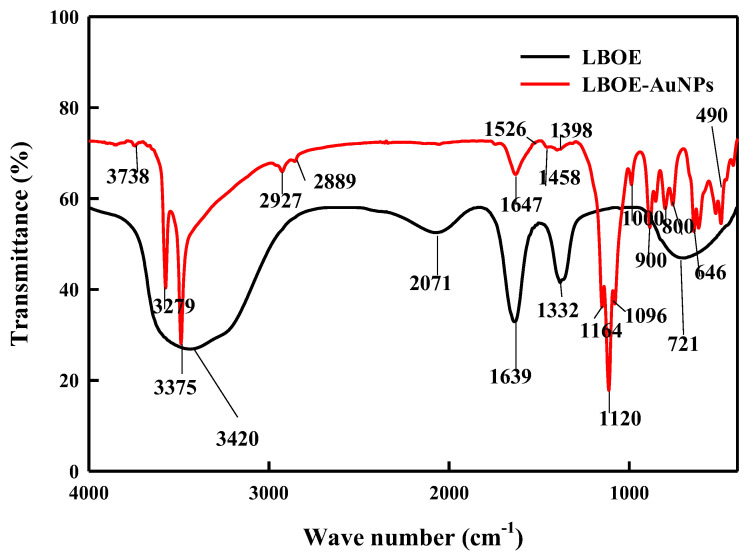
FTIR spectra of LBOE and LBOE-AuNPs.

The FT-IR spectral peaks (Figure 5) were recorded to identify and understand the possible role of bio-molecules present in the bacterial extract (LBOE) responsible for the reduction of the auric ions and capping of the bio-reduced AuNPs by the bacterial extract. The LBOE spectrum exhibited transmission peaks at 3420, 2071, 1639, 1332, and 721 cm^−1^, whereas the transmission peaks for the LBOE-AuNPs were recorded at 3738, 3279, 3375, 2927, 2889, 1647, 1526, 1458, 1398, 1164, 1120, 1096, 1000, 900, 800, 721, 646, and 490 cm^−1^.

The broad and strong bands at 3738, 3279, 3420, and 3375 cm^−1^ were assigned to hydroxyl (–OH)/amine groups (–NH) and aliphatic C–H bonding, respectively [126,127]. The stretching peaks at 2927 and 2889 cm^−1^ corresponded to the presence of linear alkane alcohols, ethers, primary and secondary amines, and C–H bond stretching vibrations in aldehydes, respectively [115,127]. The peaks at 1647, 1639, and 1526 cm^−1^ were assigned to the stretching vibrations of the C=C alkenyl group, which corresponds to –C=O due to the carboxylic acid and carbonyl group, N-H bending vibrations in primary and secondary amines, and N–O asymmetric stretching vibrations of nitro compounds [106,114,115]. The stretching peaks at 1458, 1398, and 1332 cm^−1^ were allocated to the asymmetric bending of methyl C–H bonds, symmetric stretching of –COO derived from proteins with carboxyl side groups in amino acid residues, and stretching vibrations of the C–N aromatic functional group of proteins, respectively [79,115,126,128]. The C–N stretching vibrations were observed at the peaks of 1164, 1120, 1096, and 1000 cm^−1^ [119]. The absorption peaks observed around 900 and 800 cm^−1^ were due to the absorption stretching peaks of aliphatic phosphates (P–O–C) and aliphatic choro-compounds (C–Cl), respectively [126]. The peaks at 721 and 646 cm^−1^ were found to correspond to the stretching vibrations of the thiol or thioether groups (CH_2_–S–) (C–S) [126]. Thus, these results suggest the involvement of biological molecules such as proteins, enzymes, and metabolites and functional groups (such as N–H (amine, amide) groups) in the reduction of metal salt and capping of the formed nanoparticles through the reduction process, leading to the stabilization and counterpoise of the synthesized NPs [7,8,9,10,93,113].

### 3.3. Antioxidant Activity of LBOE-AuNPs

**Figure 6:**
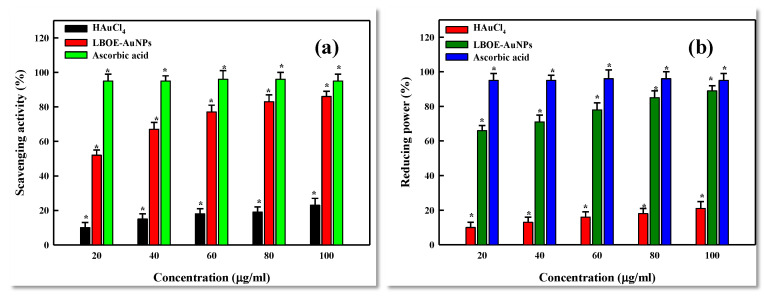
Antioxidant activity of LBOE-AuNPs. (**a**) DPPH scavenging activity, (**b**) reducing power activity. The error bars represent mean ± SD of two independent experiments performed in triplicate with * *p* < 0.05.

DPPH, a stable free-radical molecule, undergoes reduction by accepting hydrogen (H^+^) ions or electrons from AuNPs, leading to a change in the reaction color from purple to yellow when measured spectrophotometrically. The LBOE-AuNPs exhibited free radical inhibition in a dose-dependent manner (Figure 6a). The average inhibition percentage of LBOE-AuNPs was found to be in tandem with a progressive increase in the scavenging activity with increased concentrations of LBOE-AuNPs. The antioxidant activity of LBOE-AuNPs was found to be much greater than that of the bacterial extract LBOE. However, LBOE-AuNPs exhibited lower antioxidant activity as compared to the standard ascorbic acid. The highest scavenging activity was found to be 86.27% ± 3.01% at 100 μg/mL. These findings suggest that the effective capping of biomolecules on the surface of AuNPs contributes to increased free radical scavenging activity. Interestingly, the antioxidant activity of AuNPs synthesized using a cell-free extract of *L. odysseyi* has been reported for the first time. The results corroborate previous reports on biosynthesized AuNPs obtained using *Lactobacillus kimchicus* [129], *Nocardiopsis* sp. [93], *Micrococcus yunnanensis* J2 [99], and *Paracoccus haeundaensis* BC74171T [9]. Furthermore, for the measurements of reductive ability, Fe^3+^–Fe^2+^ transformations by the concentrations of LBOE-AuNPs were also investigated. Figure 6b shows the reductive capabilities of different concentrations of LBOE-AuNPs compared with LBOE and ascorbic acid (standard). The reducing power exhibited a constant increase with the increase in the concentration of LBOE-AuNPs. The uniform increase in the absorbance of the reaction mixture was an indication of an increase in the reducing power.

### 3.4. Antibacterial and Antibiofilm Activity of LBOE-AuNPs

**Figure 7:**
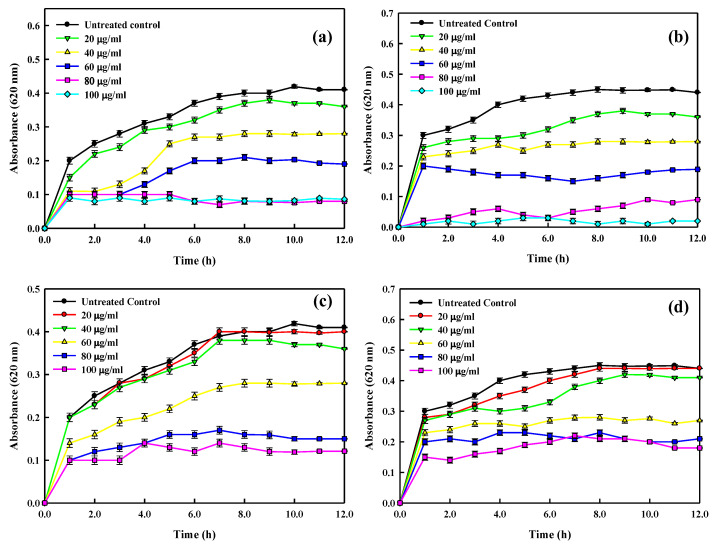
Antibacterial activity of LBOE-AuNPs against MDR bacterial growth. Changes in the absorbance (OD620 nm) of pathogenic strains at increasing LBOE-AuNP concentrations (**a**–**d**). (**a**) *E*. *coli*; (**b**) *A*. *hydrophila*; (**c**) *S*. *aureus*, and (**d**) *V*. *cholerae*.

**Figure 8:**
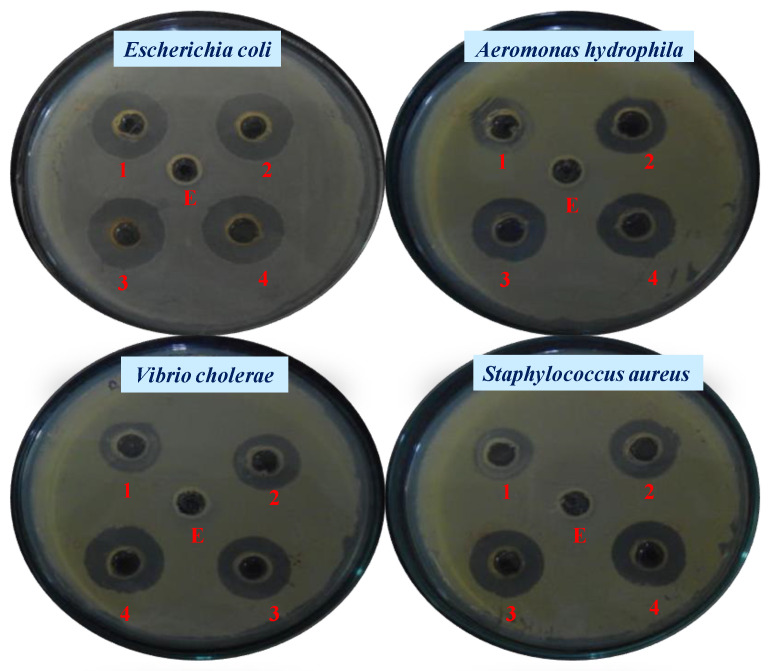
Antibacterial activity of LBOE (E) and LBOE-AuNPs at (1) 20, (2) 60, (3) 80, and (4) 100 µg/mL based on the agar well diffusion assay.

The antibacterial properties of Ag, Cu, and Zn ions, as well as zinc and copper oxides, have been proven in nanoscale materials [130]. Gold nanoparticles (AuNPs) have demonstrated particularly beneficial antibacterial action. As the least active metal, gold is non-toxic, has good biocompatibility, and has very stable chemical characteristics [131]. Additionally, gold is multivalent and can bind a variety of ligands, and AuNPs’ extremely large specific surface area offers a wealth of opportunities for interacting with target microorganisms [132]. In the present study, AuNPs fabricated using the cell-free extract of the marine bacterium *Lysinibacillus odysseyi* strain PBCW2 were evaluated for their antimicrobial activity against pathogenic strains of *E*. *coli* (EC), *S*. *aureus* (SA), *Shigella dysenteriae* (SD), *Aeromonas hydrophila* (AH), *Salmonella typhi* (ST), and *Vibrio cholerae* (VC). The MIC and MBC values for the tested strains are shown in Table 1. The results demonstrated that all pathogenic strains were invariably sensitive to the LBOE-functionalized AuNPs. The MIC and MBC values of LBOE-AuNPs were found to be in the range of 25–40 and 60–85μg/mL, respectively, suggesting promising antibacterial effects of the biosynthesized AuNPs. A similar result was also reported in *Streptomyces* sp. B5-assisted AuNPs, where an MIC value of 6.25 μg/mLwas found to be effective in arresting the growth of *Escherichia coli*, *Pseudomonasa eruginosa* and *Candida albicans* [133]. Monodispersed biogenic *C*. *albicans*-synthesized gold nanostructures inhibited the growth of *E*. *coli* and *S*. *aureus* at 128 μg/mL and 8 μg/mL, respectively [122]. The results were further substantiated with the time-kill assay (or bacterial growth inhibition) measured at an increasing concentration range of 20–100 μg/mL LBOE-AuNPs. The results shown in Figure 7 demonstrate differential growth inhibition patterns of pathogenic cells of EC, SA, AH, and VC under increasing concentrations of biosynthesized AuNPs. The results demonstrated the potent antibacterial activity of AuNPs synthesized from LBOE on the test bacterial strains at a lower concentration of 40 μg/mL. The LBOE-AuNP concentration (100 μg/mL) was found to be lethal, as no further increase in bacterial growth was noticed. However, the bacterial growth was significantly reduced to the extent of 43%, 61%, and 90% at 40, 60, and 80 μg/mL, respectively. The Gram-negative EC was observed to be more sensitive (5% survival) to the LBOE-AuNP-induced cellular damage as compared to SA (12% survival). Significant growth inhibition was observed after 2 h of LBOE-AuNP treatment. However, no further enhanced cytotoxicity was observed upon the extended incubation of up to 10–12 h. These results were further typified and confirmed by means of an agar well diffusion assay, in which varying concentrations of LBOE-AuNPs exhibited pronounced antibacterial activity against tested pathogens, as exhibited by the clear zone of inhibition around the well (Figure 8, Table 2). The antimicrobial evaluations by Abdel-Raouf et al. [134], carried out using the agar well diffusion technique, were found to be in good agreement with our results. *Galaxaura elongata* produced biogenic AuNPs with inhibitory zones against *E. coli* (17 mm), *Klebsiella pneumoniae* (17 mm), and methicillin-resistant *Staphylococcus aureus* (14 mm) [134]. Furthermore, at 50 mg/mL, the *Cladosporium cladosporioides*-assisted AuNPs showed a 12 mm inhibitory zone against *S. aureus* [135]. Antibiotic loading of azithromycin and clarithromycin on *Justicia glauca*-produced AuNPs was shown to be effective in inhibiting oral bacterial and fungal infections [87].

Various postulates have been formulated regarding the antibacterial mechanisms of AuNPs, such as (a) penetration of the cell wall through the breakdown of the latter’s components [132]; (b) the uptake of size-tailored AuNPs, i.e., smaller nanoparticles enter the bacterial cells, whereas the larger ones act on the surface to lyse and kill cells [136]; (c) surface modification variations which may also contribute to the differential killing effects of AuNPs (AuNPs primarily alter the interiors of bacterial cells, affecting the cytoderm and cyto-membranes [137]); and (d) stimulating intra-cellular imbalance, leading to the production of free radicals, which leads to detrimental killing effects on the whole cell physiology [138].

**Figure 9:**
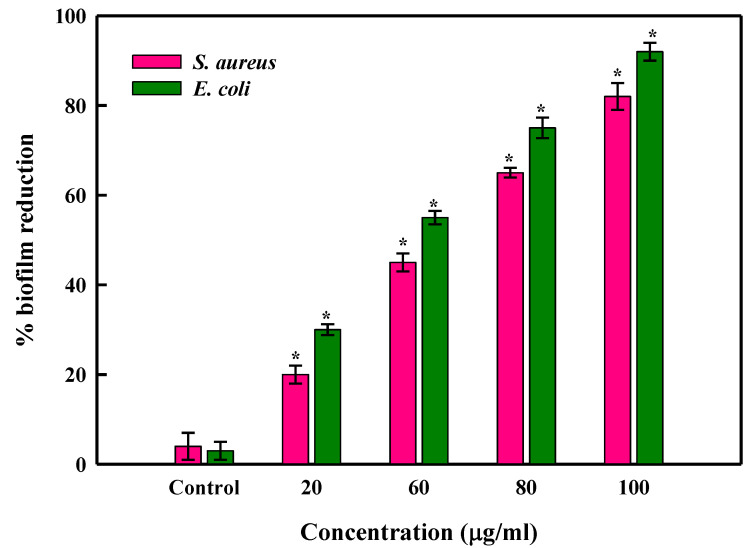
Dose-dependent reduction in bacterial biofilm formation under different LBOE-AuNP concentrations. The error bars represent the mean ± SD of two independent experiments performed in triplicate with * *p* < 0.05 vs. control.

Successful medical treatments have become more difficult to obtain due to the increasing pervasiveness of antibiotic resistance and inefficacy when drug-resistant bacteria are involved in chronic and life-threatening infections. The antibiofilm activity of LBOE-AuNPs was assessed via a crystal violet assay by treating the test strains with varying concentrations of AuNPs on sterile microtiter plates. The inhibition levels of biofilms formed by biofilm-forming cells of SA and EC were found to be 82% ± 3% and 92% ± 2% at 100 μg/mL LBOE-AuNPs (Figure 9), respectively, after 24 h of treatment. In order to restrict the growth of biofilms, antimicrobials must be able to penetrate the polysaccharide matrix to obtain transient accessibility to embedded microbial cell populations. Earlier studies demonstrated the anti-biofilm efficacy of GA-AgNPs against MβL- and EsβL-producing strains, in which GA-AgNPs easily penetrated and reduced biofilm formation. Similar conclusions were also reported by Kalishwaralal et al. [139] against the biofilm aggregates formed by *Staphylococcus epidermidis* and *Pseudomonas aeruginosa*, where 100 nM AgNPs resulted in 95–98% biofilm reductions. Ansari et al. [140] reported a reduction (95%) in the biofilm formation formed by the clinical isolates of *Klebsiella* sp. and *E*. *coli* at 50 μg/mL AgNPs. Indeed, studies on the antimicrobial activity of nanoparticles (NPs) suggest variable responses to the types and nature of NPs [141]. Baek and An [142] found that Gram-negative bacteria such as E. coli are more sensitive to NPs than gram-positive bacteria such as *S. aureus* and *B. subtilis* [143]. Antibiotics and NPs are more vulnerable to fastidious bacteria than slow-growing bacteria [144]. Tolerance exhibited by slow-growing bacterial strains is likely linked to the genomic expression of stress-response genes [145]. As a result, the antibacterial properties of NPs differ depending on the bacterial species and strain. In the presence of NPs, the structure of the cell wall plays a key role in bacterial antibiotic resistance or susceptibility.

**Figure 10:**
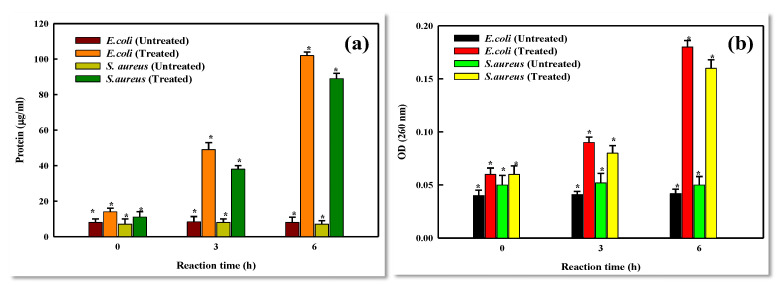
Effect of LBOE-AuNPs on (**a**) protein leakage and (**b**) nucleic acid leakage in reference to treated bacterial cells as compared to control cells. The error bars represent the mean ± SD of two independent experiments performed in triplicate with * *p* < 0.05.

The measurements of protein leakage of test bacterial cells treated with LBOE-AuNPs were found to be the same as those of controls (untreated cells) at the commencement of the experiment. Subsequently, after 3 h of treatment, the treated cells seeped out 38–49 μg/mL protein in comparison to untreated cells. Furthermore, after6 h of the incubatory period, the LBOE-AuNP-treated cells exhibited about four-times-higher leakage levels as compared to controls. The cells of *E. coli* exhibited a higher quantity of protein leakage (102 μg/mL) than *S. aureus* (89 μg/mL) after 6 h of treatment (Figure 10a). A similar trend was also exhibited in the case of nucleic acid leakage, wherein the seepage of intracellular materials, post-exposure to LBOE-AuNPs at varied time intervals, was recorded via absorbance measurements at 260 nm (OD_260_). Initially, the nucleic acid leakage level was almost the same for both control/untreated and LBOE-AuNPs treated cells. Furthermore, an augmented liberation of nucleic acids (noticeable more marked in *E. coli* after 3 and 6 h treatment with AuNPs) was observed as compared to controls (Figure 10b).

### 3.5. DNA Interaction Study

**Figure 11:**
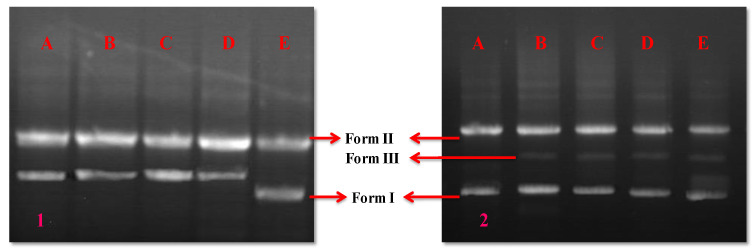
Cleavage patterns with different concentrations of (1) HAuCl_4_ and (2) LBOE-AuNPs. (A) Control, (B) 20 μg/mL, (C) 60 μg/mL, (D) 80 μg/mL, (E) 100 μg/mL.

For DNA interaction investigations with biomimetically produced LBOE-AuNPs and HAuCl_4_, the cleavage of vector pBR322 DNA was investigated using agarose gel electrophoresis (Figure 11a). In the control sample (devoid of the LBOE-AuNP complex), two clear bands were observed in lane 1, which can be attributed to the relatively fast migration of the intact supercoil form (Form I) versus the slower moving migration of the open circular form (Form II), the latter of which was generated from the supercoiled form when a DNA nick occurred on one of its strands. The supercoiled Form I DNA was wrecked and broken when the vector DNA interacted with high quantities of LBOE-AuNPs, resulting in an increase in the intensity of Form II DNA, which may be attributed to the binding of LBOE-AuNPs to the DNA, lowering Form I mobility in contrast to the control sample. Additionally, nicking the supercoiled Form I at the measured concentrations of Au^3+^ ions was shown to enhance the intensity of Form II DNA, resulting in the formation of a third band of linear Form III DNA (Figure 11b). No noteworthy effect on vector DNA was ascertained with increased concentrations of HAuCl_4_ (Figure 11c). Furthermore, Au^3+^ ions caused some breaks on the DNA strand, acting as a chemical nuclease with the LBOE-AuNPs crafting nicks and breaks on the DNA strand, thus decelerating its mobility via site binding. According to Duman et al. [86], the molecules travel in the gel depending upon the function of their respective charge, mass, size, and shape, in which the DNA forms a supercoiled state migrating/drifting faster than the open circular molecular state of the same mass and charge. A similar result was proposed by Duman et al. [86] in the case of *Matricaria chamomilla*-fabricated copper oxide nanoparticles (CuO-NPs), establishing the effect of CuO-NPs on the DNA breakage of plasmid vector pBR322. In contrast, Raju et al. [146] reported that plasmid DNA coupled with AuNPs was unable to move from the sample well, specifying that the plasmid DNA binding to AuNPs was due to charge attraction, i.e., the negatively charged DNA molecule preferentially binds to the positively charged NPs.

### 3.6. Dye Degradation Study

**Figure 12:**
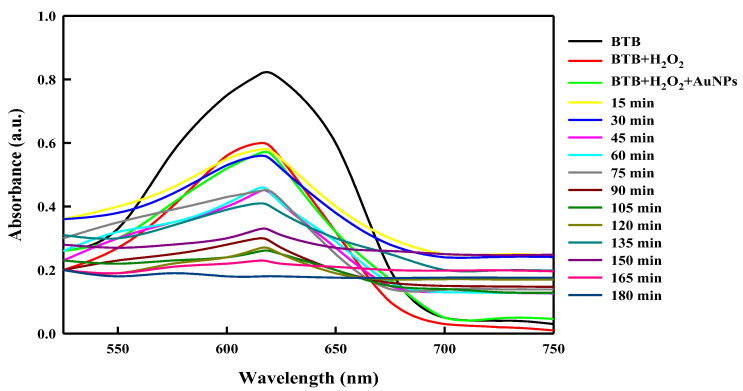
UV–visible spectral analysis of bromothymol blue (BTB) degradation in the presence of LBOE-AuNPs with reference to time.

**Figure 13:**
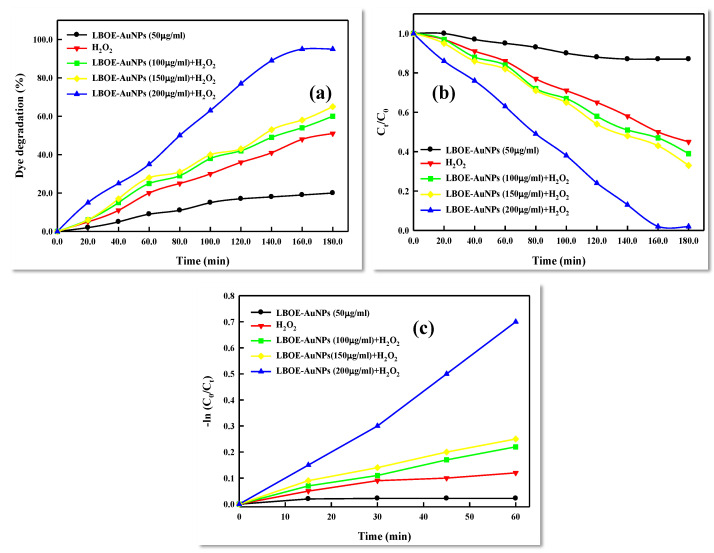
(**a**) Percentage analysis of dye (BTB) degradation at various concentrations of LBOE-AuNPs with H_2_O_2_; (**b**) comparison of photocatalytic dye degradation efficiency for BTB in the presence of LBOE-AuNPs+H_2_O_2_; and (**c**) reaction kinetics of the photocatalyzed degradation of BTB in the presence of LBOE-AuNPs+H_2_O_2_.

The photocatalytic activity of *Lysinibacillus odysseyi* PBCW2-extract-mediated green synthesized AuNPs was studied by monitoring the kinetics of the degradation pattern of BTB (bromothymol blue) dye, used as a model aquatic pollutant that is resistant to direct oxidation. The degradation steps were followed by a free radical reaction, carried out using hydrogen peroxide (H_2_O_2_) and LBOE-AuNPs and inspected through a time-dependent observation of the absorbance spectrum of the reaction solution (Figure 12). The intense peak of BTB observed at an absorbance (λ_max_) of 616 nm decreased significantly in the presence of LBOE-AuNPs (with or without H_2_O_2_) under sunlight irradiation. The dye-degrading efficiency of LBOE-AuNPs (50 μg/mL) without H_2_O_2_ was found to be moderately efficient, whereas that of H_2_O_2_ showed a degradation efficiency of 63.04% at 3 h of irradiation (Figure 13a). Furthermore, a remarkable degradation efficiency was observed in the conjugation of LBOE-AuNPs + H_2_O_2_, which increased with a simultaneous increase in the concentration of nanoparticles. The use of LBOE-AuNPs (100, 150, and 200 μg/mL) with H_2_O_2_ results in 59.68%, 69.25%, and 91.03%, respectively, after 2 h of illumination. Subsequently, at 3 h of irradiation, the dye was fully degraded, indicating the photocatalytic debasement of dye molecules by LBOE-AuNPs. The reaction kinetics of LBOE-AuNPs illustrates a dye model reaction as pseudo-first-order kinetics with the measurements of the rate constant of dye degradation by LBOE-AuNPs (100, 150, 200 μg/mL with H_2_O_2_) and H_2_O_2_ alone as 0.005200, 0.005509, 0.01131, and 0.003766 min^−1^, computed using the linear relationship of ln(C_o_ − C_t_/C_o_) vs. time (Figure 13b,c). The quotient of reusability of LBOE-AuNPs for photo-irradiation catalyzed BTB dye degradation was examined for six cycles, with results exhibiting the stability of LBOE-AuNPs after six cycles, and indicating a degradation efficiency of 91%. The rationale for rapid BTB degradation may be ascribable to the generation of OH^−^ ions and electronic adsorption from stable dye molecules, effectively elicited by LBOE-AuNPs, owing to their nano-scale surface properties [147].

## 4. Conclusions

In the present study, we investigated the use of a green, economical, biological reducing agent, *Lysinibacillus odysseyi* extract, for the biosynthesis of gold nanoparticles (AuNPs) through a green chemistry method, developed through comprehensive process optimization and standardization. The appearance of a ruby red color was the preliminary confirmation of the formation of AuNPs, and this was quantified by analyzing the UV–vis spectrum exhibiting a surface plasmon peak at 520 nm. The XRD studies confirmed the face-centered cubic lattice and crystalline nature of the synthesized biogenic AuNPs. TEM, SEM, SAED, DLS, TGA, FT-IR, and zeta potential analysis were also carried out to substantiate the synthesis of AuNPs. The potential biological activities of antioxidants, antibacterials, DNA cleavage, and dye degradation were assiduously and methodically corroborated by means of various standardized assays. These multifarious activities lead us to recommend biogenic LBOE-AuNPs as “prospective benefactors” in prophylactic and therapeutic applications against human bacterial pathogens, and they possess striking catalytic efficacy in the reduction of BTB dye.

## Figures and Tables

**Table 1:** MIC and MBC values of LBOE-AuNPs against pathogenic strains.

S. No.	Pathogenic Strains	LBOE-AuNPs (µg/mL)	
		MIC	MBC
1.	*Aeromonas hydrophila*	40 ± 1.2	80 ± 1.4
2.	*Escherichia coli*	25 ± 0.8	60 ± 0.9
3.	*Staphylococcus aureus*	35 ± 1.0	85 ± 1.2
4.	*Vibrio cholerae*	30 ± 0.9	70 ± 0.8

**Table 2:** Antibacterial activity of varied concentrations of LBOE-AuNPs against pathogenic strains (zone of inhibition measured in mm).

S. No.	Pathogenic Strains	LBOE	LBOE-AuNPs (µg/mL)			
			20	60	80	100
**1.**	*Escherichia coli*	0	19 ± 1.2	21 ± 0.7	23 ± 0.8	26 ± 1.0
**2.**	*Vibrio cholerae*	0	13 ± 1.0	14 ± 1.1	18 ± 0.7	20 ± 0.8
**3.**	*Staphylococcus aureus*	0	10 ± 0.8	15 ± 1.0	18 ± 1.1	20 ± 1.0
**4.**	*Aeromonas hydrophila*	0	08 ± 1.0	11 ± 0.9	13 ± 0.7	15 ± 1.2

## Data Availability

The data generated in the present study can be shared upon the request of the authors.

## Supplementary Materials

The following supporting information can be downloaded at: https://www.mdpi.com/article/10.3390/nano12172940/s1, Figure S1: Particle histogram in TEM image.
