# Supplementary material for: Green Chemistry Based Gold Nanoparticles Synthesis Using the Marine Bacterium Lysinibacillus odysseyi PBCW2 and Their Multitudinous Activities
Source: Nanomaterials (Basel). 2022 Aug 26;12(17):2940. doi: 10.3390/nano12172940 (PMC9458051; doi:10.3390/nano12172940)
Supplement: Supplementary file 1 [file nanomaterials-12-02940-s001.zip › nanomaterials-1858471-supplementary.pdf]

Supplementary Materials

Green Chemistry Based Gold Nanoparticles Synthesis Using the Marine Bacterium *Lysinibacillus odysseyi* PBCW2 and Their Multitudinous Activities

Tijo Cherian *, Debasis Maity, Ramasamy T. Rajendra Kumar, Govindasamy Balasubramani, Chinnasamy Ragavendran, Suneelkumar Yalla, Raju Mohanraju and Willie J. G. M. Peijnenburg *

* Correspondence: tvarghese891@gmail.com (T.C.); willie.peijnenburg@rivm.nl (W.J.G.M.P.)

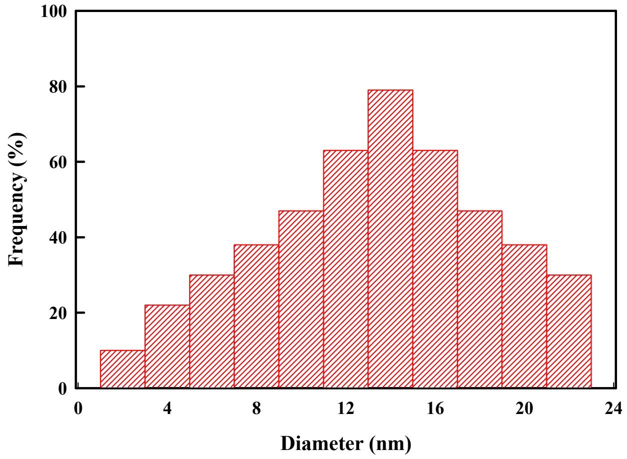

Figure S1. Particle histogram in respective TEM image.
